# Unmet needs and behaviour during the Ebola response in Sierra Leone: a retrospective, mixed-methods analysis of community feedback from the Social Mobilization Action Consortium

**DOI:** 10.1016/S2542-5196(20)30008-5

**Published:** 2020-02-26

**Authors:** Laura A Skrip, Jamie Bedson, Sharon Abramowitz, Mohammed B Jalloh, Saiku Bah, Mohamed F Jalloh, Ollin Demian Langle-Chimal, Nicholas Cheney, Laurent Hébert-Dufresne, Benjamin M Althouse

**Affiliations:** Institute for Disease Modeling, Bellevue, Seattle, WA, USA (L A Skrip PhD, B M Althouse PhD); Restless Development Sierra Leone, Freetown, Sierra Leone (J Bedson MSSc, S Bah MSc); Consultant to the Bill & Melinda Gates Foundation, Seattle, WA, USA (J Bedson); Consultant to UNICEF, New York, NY, USA (S Abramowitz PhD); FOCUS 1000, Freetown, Sierra Leone (M B Jalloh MPH, M F Jalloh MPH); Vermont Complex Systems Center, Department of Computer Science, University of Vermont, Burlington, VT, USA (O D Langle-Chimal MSc, N Cheney PhD, L Hébert-Dufresne PhD); Département de Physique, de Génie Physique, et d’Optique, Université Laval, Québec City, QC, Canada (L Hébert-Dufresne); Information School, University of Washington, Seattle, WA, USA (B M Althouse); and Department of Biology, New Mexico State University, Las Cruces, NM, USA (B M Althouse)

## Abstract

**Background:**

The west African Ebola epidemic (2014–15) necessitated behaviour change in settings with prevalent and pre-existing unmet needs as well as extensive mechanisms for local community action. We aimed to assess spatial and temporal trends in community-reported needs and associations with behaviour change, community engagement, and the overall outbreak situation in Sierra Leone.

**Methods:**

We did a retrospective, mixed-methods study. Post-hoc analyses of data from 12 096 mobiliser visits as part of the Social Mobilization Action Consortium were used to describe the evolution of satisfied and unsatisfied needs (basic, security, autonomy, respect, and social support) between Nov 12, 2014, and Dec 18, 2015, and across 14 districts. Via Bayesian hierarchical regression modelling, we investigated associations between needs categories and behaviours (numbers of individuals referred to treatment within 24 h of symptom onset or deaths responded to with safe and dignified burials) and the role of community engagement programme status (initial *vs* follow-up visit) in the association between satisfied versus unsatisfied needs and behaviours.

**Findings:**

In general, significant associations were observed between unsatisfied needs categories and both prompt referrals to treatment and safe burials. Most notably, communities expressing unsatisfied capacity needs reported fewer safe burials (relative risk [RR] 0·86, 95% credible interval [CrI] 0·82–0·91) and fewer prompt referrals to treatment (RR 0·76, 0·70–0·83) than did those without unsatisfied capacity needs. The exception was expression of unsatisfied basic needs, which was associated with significantly fewer prompt referrals only (RR 0·86, 95% CrI 0·79–0·93). Compared with triggering visits by community mobilisers, follow-up visits were associated with higher numbers of prompt referrals (RR 1·40, 95% CrI 1·30–1·50) and safe burials (RR 1·08, 1·02–1·14).

**Interpretation:**

Community-based development of locally feasible, locally owned action plans, with the support of community mobilisers, has potential to address unmet needs for more sustained behaviour change in outbreak settings.

**Funding:**

Bill & Melinda Gates, Bill & Melinda Gates Foundation, and National Institutes of Health.

## Introduction

In the 2014–15 west African outbreak of Ebola virus disease, local populations were confronted with decisions on how to handle the sick and deceased. Initial public health recommendations from international authorities were based on experiences with previous outbreaks of limited scale in central Africa, and were not adapted to reflect local ecological, sociocultural, and public health contexts. Conflicts between these recommendations and local customs, as well as a lack of understanding about Ebola and its transmission, generated widespread community-level concerns.^1,2^ Moreover, discontent with response measures was compounded by high degrees of unfulfilled basic needs, distrust in authority, and fragile health and economic systems, with the three most affected countries ranking among the lowest 8% globally on the Human Development Index.^3,4^

Observed behaviours during the initial phases of the Ebola outbreak throughout west Africa ranged from individuals looting treatment centres, to families hiding the sick, to community-level rejection of safe burial practices. While these behaviours were counterproductive to transmission reduction, they were regarded by local populations as reasonable reactions to the intensified control or unexplained intervention exerted in a top-down manner. In addition, such behaviours might have been driven by underlying mistrust of government authorities. By the end of 2014, sufficient funding and personnel had been released in Sierra Leone to ensure that response activities were informed by a social-behavioural sciences approach that prioritised community engagement, social mobilisation, and local ownership and empowerment. This was a transformative policy approach, and because it correlated with the epidemic’s decline, has been perceived to have facilitated the reduction in acts of direct violence and overt avoidance towards the public health response.^5^

**Figure 1:**
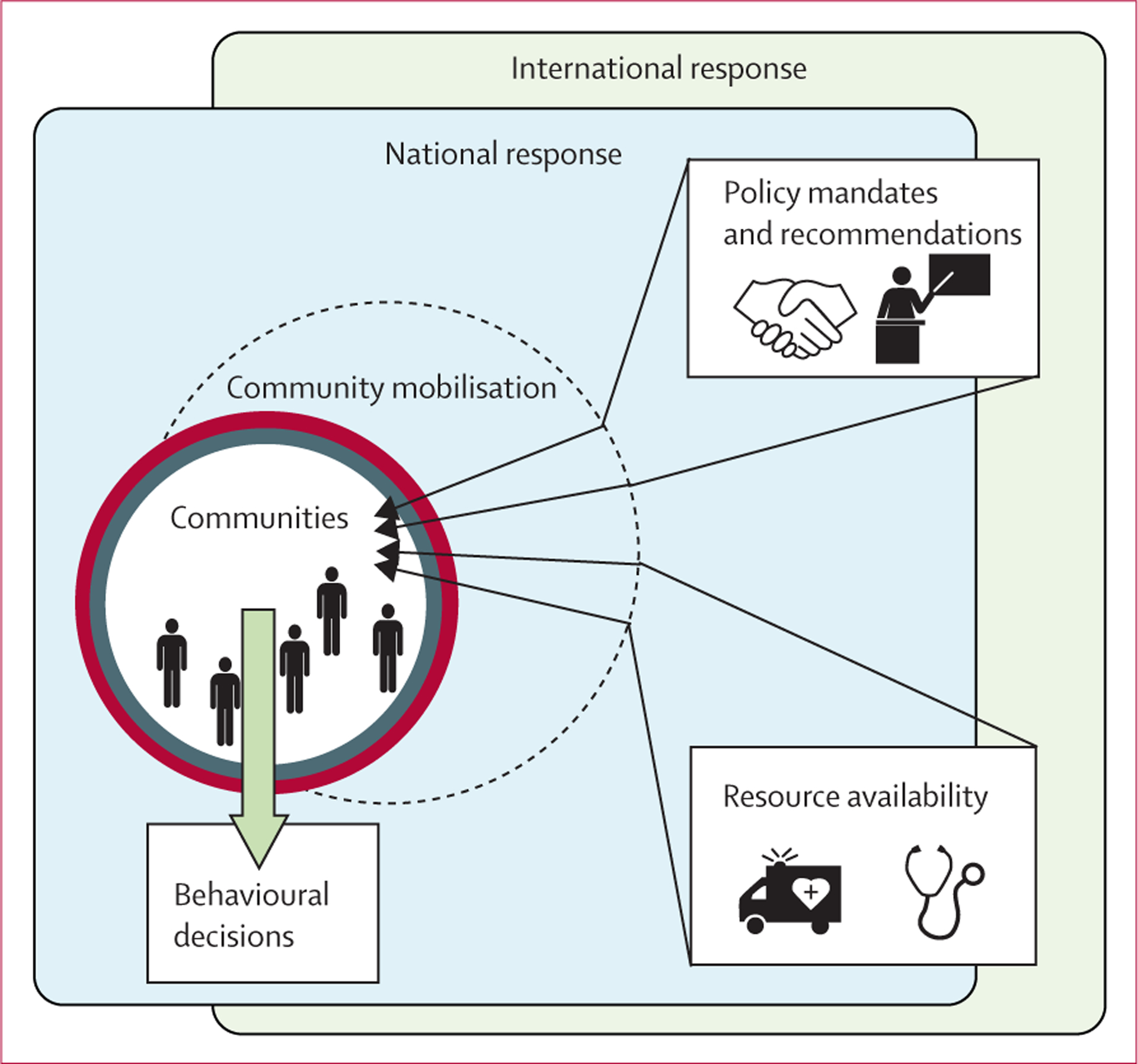
Ecological framework for behaviour change in an epidemic setting The evolution of needs during an outbreak is influenced by national and international policies as well as resource availability. That is, multiscale components of outbreak response are expected to affect the exaggeration and satisfaction of needs at the community level. Unmet needs affect the adoption of prescribed behaviour change recommendations. Structured participatory approaches (such as the Community-led Ebola Action methodology) that underpin community engagement provide a mechanism for promoting autonomy and guiding resource allocation to address needs for improved adoption of public health behaviours in accordance with or in addition to recommendations. The effectiveness of community mobilisation is therefore related to the availability of resources and needs-supportive policies across scales.

Decisions on whether or not to act on messages of public health campaigns are influenced by the level of autonomous motivation to undertake behaviour change.^6–8^ According to self-determination theory,^9^ engagement in extrinsically motivated behaviours, such as participation in outbreak response efforts that are counter to personally or culturally satisfying practices, or both (eg, bushmeat consumption, traditional burials), would be increasingly enhanced and maintained as people identify with their value.^10^ In an outbreak setting, community engagement and social mobilisation efforts have the potential to facilitate the internalisation of public health regulations by transforming the human ecology for health-related social behaviours at the local or community level (figure 1).

Community-based efforts create environments characterised by autonomy as local actors decide how they will address the outbreak, social acceptance as behaviour changes are made collectively, and increased understanding and awareness of an unfamiliar situation. These three components align with human needs for autonomy, relatedness, and competence, and together with needs for basic physical goods (eg, food and drinking water), security, and safety, have been widely considered as universal and fundamental in influencing wellbeing and behaviour.^10,11^ While some outbreak-setting needs were addressed within communities, others warranted a trusted link between local entities and other levels of the response. Community mobilisers served that role. Their engagement with communities functioned to promote multidimensional needs satisfaction and thus catalyse the adoption and utilisation of policies and resources, respectively, that were otherwise perceived as inaccessible, untenable, or inflexible.

In Sierra Leone, the Social Mobilization Action Consortium (SMAC) trained and supported a network of nearly 2500 community mobilisers in the implementation of the Community-led Ebola Action (CLEA) approach.^12,13^ During initial visits to communities, mobilisers used structured tools to facilitate community inquiry in a single triggering event, attended by a representative group of community members, to create a sense of urgency and call to action in communities. Triggering events were done to support communities to conduct their own analysis and take their own action to prevent disease transmission, promoting self-ownership and community agency (table). The aim was to identify Community Champions as key focal points and to support communities in the development of plans focused on action to end Ebola transmission by facilitating and normalising risk-reduction behaviours. Subsequent visits to communities were made to follow the progress of these plans and provide updated information and support. Information collected during visits afforded an opportunity to explore the role of competing issues, from hunger to disrespect to school closures to teenage pregnancy, and their resolution on influencing local behaviours.

Although previous work has emphasised the importance of engaging communities for positive behavioural change in outbreak response,^15–18^ the analysis of large-scale data to explore an ecological model in supporting the use and understanding the effectiveness of community engagement has not been done. We retrospectively assessed spatial and temporal trends in the expression of community-level concerns during the 2014–15 Ebola outbreak in Sierra Leone. We investigated the association between needs satisfaction and behaviours to reduce transmission (namely, referral to treatment for the seriously sick and safe burial for the deceased), to provide initial empirical evidence for an underlying mechanism of behaviour change, in general and resulting from community engagement specifically, in complex ecologies during outbreaks of emerging infectious disease.

## Methods

### Data collection

This study was a mixed-methods, retrospective analysis of previously collected data. Data were obtained from reports made by SMAC community mobilisers for triggering events and subsequent follow-up visits to approximately 12 000 communities in Sierra Leone. Follow-up visits generally occurred once every 3 weeks after the triggering event, apart from in Western Area where they could occur as frequently as once a week after the triggering event. Earlier follow-up visits occurred if mobilisers thought them necessary because of questions or concerns expressed by the community. Information collected in the reports included geographical identifiers, numbers of individuals participating in the community visits by mobilisers, numbers of seriously sick and deceased individuals in the past week, and numbers of prompt referrals to treatment and safe burials in the past week. The reports also included open-ended responses to questions about the community’s Ebola-related concerns at the time of the visit. Data were collected via paper and digital forms, with digital reporting used by five districts only. Community mobilisation occurred between Nov 12, 2014, and Dec 18, 2015. As described elsewhere,^12^ community mobilisers were youth workers (aged 18–28 years) who had previously been involved in an HIV/AIDS community programme coordinated by Restless Development in Sierra Leone. Mobilisers underwent training in the CLEA approach as well as in data collection and submission via standardised forms.^19^

### Categorisation of needs

For the paper reports, data for the analysis were derived from a question about the most common Ebola-related concern expressed by the community (ie, mobilisers’ documentation of “What are the most commonly expressed Ebola-related concerns expressed by community members?”). For the digitally submitted reports, data were derived from the question about the most common concern (precategorised on the form into nine concerns), open-ended questions on concerns related to burial and treatment practices, and an open-ended question about the biggest change in the community. In the digital dataset, the nine concern categories included the option for multiple selection and were specified as “delay in picking up dead bodies by burial team”, “unprofessionalism of the burial team”, “survivors are spreading Ebola through sexual contact”, “orphans needing care”, “discrimination against survivors”, “closing of schools”, “re-opening of schools”, “lack of food in quarantined homes”, and “other”. All forms for data collection and data codebooks are publicly available.^19,20^

Open-ended qualitative responses in the mobilisers’ reports were coded to identify common themes (appendix pp 10–14). Individual responses could be assigned multiple codes. The themes were then mapped to a pre-established needs classification scheme proposed by Tay and Diener^14^ (table) using an inductive approach.^19^ Similarly, the concerns categories from the digital forms were mapped to the classification scheme.

The classification represented a combination of theories of motivation. The Tay and Diener scheme was extended to include a capacity category based on findings from the thematic analysis. Entries were also coded according to whether communities were communicating unsatisfied or satisfied needs (table).

A subset of approximately 5000 entries was manually coded (author LAS). A linear support vector machine (SVM) was implemented to automatically code remaining entries. The use of a supervised learning approach to text classification allowed for training and evaluation of the SVM model, as well as generation of predictive probabilities for each result. Spot-checking of the classification results ensured that all possible codes for an entry were included and that the sentiment was captured to determine if the communities were expressing needs unsatisfied or satisfied. Approximately 75% of the automatically classified entries were manually checked.

Analysis of word frequency per category was used to assess consistency of results with intended classification. Topic modelling was also done to determine consistency between naturally emerging clusters from the data and the predefined classification scheme (appendix pp 3–6, 15–16).

### Spatial, temporal, and spatiotemporal analyses

Spatial and temporal trends in the expression of needs were evaluated. For temporal trends, data were aggregated weekly. For spatial trends, data were aggregated according to district level and province level: Eastern Province (Kailahun, Kenema, and Kono districts), Western Area (Western Area Rural and Western Area Urban), Southern Province (Bo, Bonthe, Moyamba, and Pujehun districts), and Northern Province (Bombali, Kambia, Koinadugu, Port Loko, and Tonkolili districts). Trends were visualised as the prevalence of the needs category, or the proportion of communities reporting a concern in a given needs category out of all communities visited in a unit of time or space, or both time and space. χ^2^ tests were used to determine significant differences in frequencies of reported needs categories across units of time and space. Post-hoc comparisons were made with adjustment by the Bonferroni correction to further investigate any statistically significant general trends (p<0·05).

Because of varying levels of data collection across different space-time units, we generated country-wide surfaces for the prevalence of expressed needs categories at the district level via spatiotemporal area-to-point kriging. As described above, prevalence was calculated as the number of communities expressing a particular needs category divided by the total number of communities per space-time unit. Results were used to relate ecological factors of resource availability and behaviour-related policy to trends in community needs expression.

### Statistical analysis

Associations between behaviours and the needs classification categories were investigated using hierarchical Bayesian count regression modelling. A full model description is presented in the appendix (p 1). For each model, the outcome was either count of individuals referred to treatment within 24 h of symptom onset or count of deaths responded to with safe and dignified burials, as reported for the week preceding a mobiliser’s visit. Covariates included the six unsatisfied needs categories, the six satisfied needs categories, and expression of mastery, and their coefficients were interpreted as relative risks (RRs). All covariates were treated as binary (ie, 0 for no expression of the category and 1 for expression of the category at a given visit). In addition, an offset term was included to account for the number of reported events warranting action. For the outcome of treatment referrals, the offset was the number of seriously sick individuals in the community during the reporting period; for safe burials, the offset was the number of deaths in the community during the reporting period. The outcomes were expected to be proportional to these numbers, which were in turn assumed to be indicative of outbreak severity, since reporting to SMAC mobilisers would be representative of reporting to the overall response. The parameter of each offset term was set to 1. Overdispersion in the counts of referrals and safe burials was addressed by considering the fit of the negative binomial as well as Poisson model to each of the outcomes. Models were compared for each outcome on the basis of the deviance information criterion and Watanabe-Akaike Information Criterion.

A separate regression analysis was done to establish the relation between visit type (triggering *vs* follow-up) and behavioural outcomes (count of prompt referrals or count of safe burials), after controlling for epidemic week. Poisson and negative binomial models were considered, as specified for the needs analysis. Results were reported as RRs, based on the coefficient for visit type in each of the two models. Additionally, hierarchical Bayesian logistic regression was done to assess the association between visit type and needs categories, after controlling for epidemic week. Odds ratios (ORs) for each needs category, six satisfied, six unsatisfied, and mastery, were generated.

For all statistical models, district-level random intercepts were included to account for shared contextual factors among communities within the same district, and distinct contextual factors between communities from different districts. For the final log-linear models, coefficients with 95% credible intervals (95% CrIs) that did not span zero were considered significant. Coefficients were exponentiated for interpretation as RRs or ORs. Analyses were done with R version 3.5.2. The raw dataset (of which the analysis dataset was a subset) is available in a public repository.^20^

### Role of the funding source

The funders of the study had no role in study design, data collection, data analysis, data interpretation, or writing of the report. All authors had full access to all the data in the study and the corresponding author had final responsibility for the decision to submit for publication.

## Results

12 096 community visits (6361 triggering and 5735 follow-up) from Nov 12, 2014, to Dec 18, 2015, were included in the descriptive analysis. Communities from 120 of the 153 chiefdoms in Sierra Leone were represented. Nearly half (n=15) of the unrepresented chiefdoms were in Kenema District. Word frequency analysis suggested that the results of classification were consistent with the intended meaning of each needs category (table, appendix p 2). Topic modelling showed that responses tended to naturally cluster according to the needs categories with probabilities substantially higher than chance alone, offering validation to the classification (appendix pp 3–6, 15–16). Among all observed combinations of needs categories expressed at a given community visit, the three most frequently reported scenarios involved expression of unsatisfied security needs (1985 [18·2%] of 10 904 visits at which at least one needs category was expressed), mastery (1712 [15·7%]), and unsatisfied capacity needs (1509 [13·8%]). Of 10 904 visits in which any concerns were expressed, 8078 (74·1%) involved concerns that mapped to one needs category, while 2826 involved concerns that spanned multiple categories.

**Figure 2:**
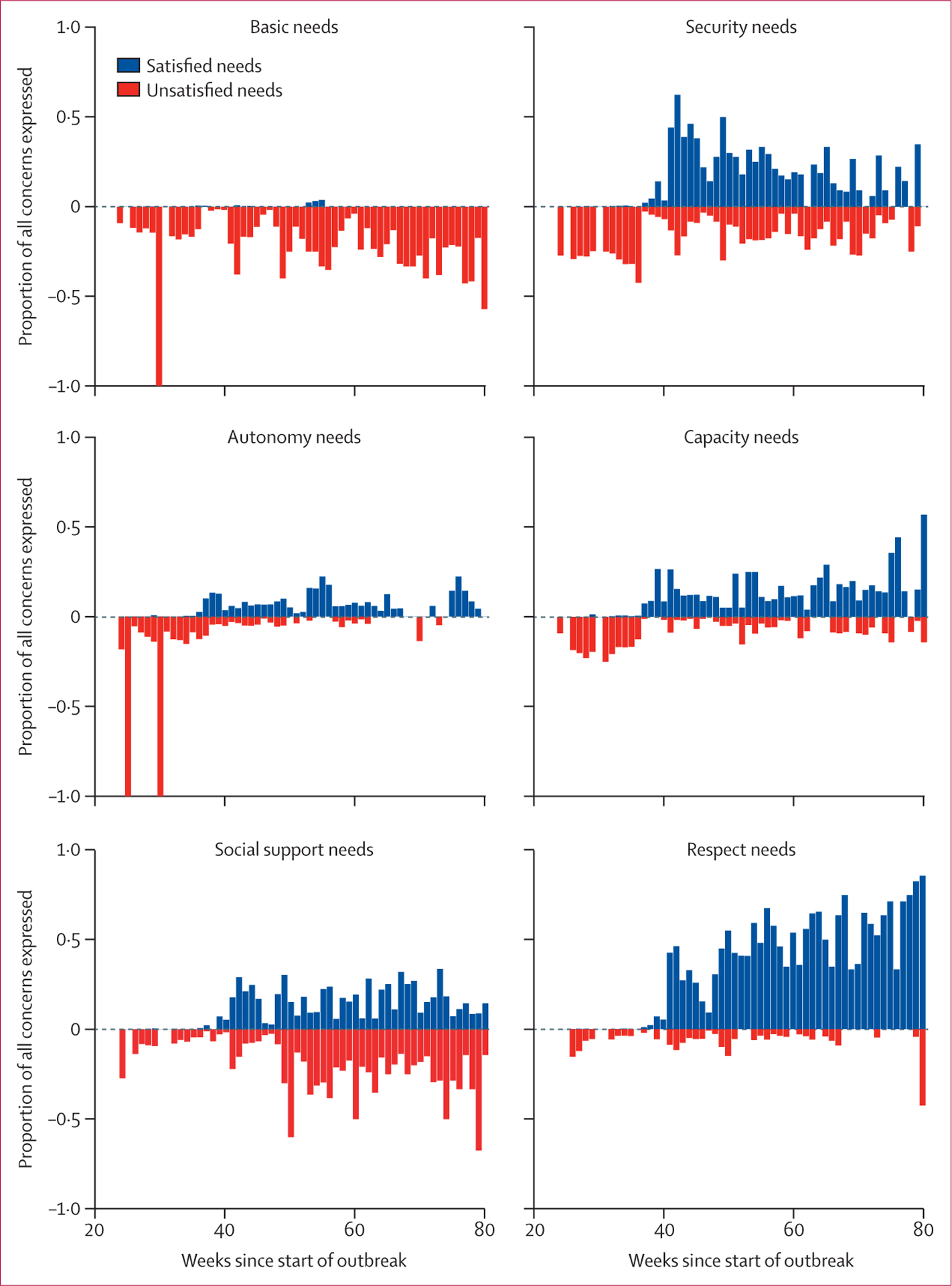
Weekly trends in expression of needs, relative to the start of the Ebola outbreak in Sierra Leone Blue bars represent weekly prevalence of needs satisfaction; red bars represent prevalence of expression of unsatisfied needs.

The prevalence of unsatisfied autonomy and capacity needs tended to be higher within the first 3 months of data collection (Nov 12, 2014, to Feb 9, 2015) than the subsequent time period (figure 2). Reports from the first 3 months were predominantly from triggering visits (appendix p 7). Unsatisfied basic needs were more consistently expressed over the course of the outbreak (mean weekly prevalence 20·6%), whereas unsatisfied social support needs became more prevalent during the second period (mean weekly prevalence 23·0%) than the first period (mean weekly prevalence 7·5%).

Reports involving needs satisfaction were not common until February, 2015, or week 36 of the outbreak in Sierra Leone. Satisfaction of respect and security needs was more prevalent than that of other needs categories. Respect was increasingly expressed over time. Although more prevalent than other needs categories, satisfaction of security needs peaked in March, 2015 (week 42 of the outbreak), and subsequently decreased during the course of data collection. Mastery was expressed by the majority of communities visited after week 36 of the outbreak (mean weekly prevalence 59·9%).

**Figure 3:**
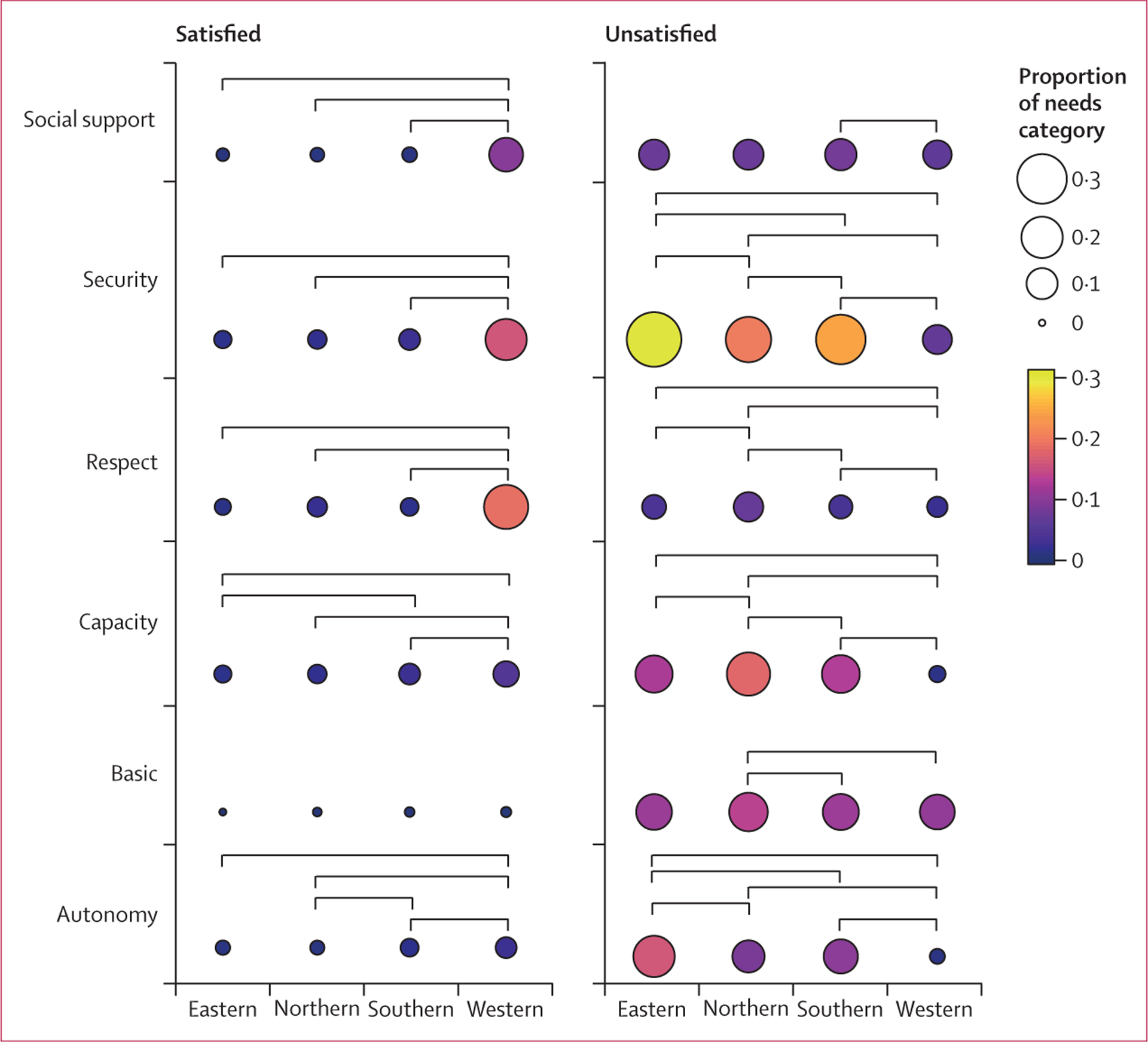
Province-level pairwise comparisons for the frequency of expression of each satisfied and unsatisfied needs category Statistical significance was based on the p value from pairwise χ^2^ test after Bonferroni adjustment. Colour and size reflect proportion of needs category for a given province out of all needs expressed in that province. Lighter colours (from purple to yellow) and larger diameter circles represent more reporting of a given needs category, relative to other categories, in a given province. Each pairwise comparison that was significant (p<0·05 after Bonferroni adjustment) is represented by a link between corresponding provinces.

The greatest number of community visits occurred in the Northern Province where the highest number of Ebola cases was reported (5630 [46·5%] of 12 096 visits) and the fewest occurred in the Western Area (1265 [10·5%]; appendix p 8). On average, concerns fell into 1·1 needs categories per visit in the Eastern Province (1709 of 1592 visits), Southern Province (3837 of 3609), and Northern Province (6218 of 5630), whereas visits in the Western Area were each associated with 3·2 categories (4062 of 1265). The expression of mastery differed significantly across provinces, with significantly less mastery reported in the Eastern Province than in the other three provinces (all pairwise p<0·0001 after Bonferroni adjustment). The Northern Province had higher proportions of unsatisfied capacity and respect needs than all other provinces (figure 3). All pairwise comparisons for unsatisfied security needs were significant, with the Eastern Province having the highest proportion of unsatisfied security needs (521 [30·5%] of 1709 categorised needs expressed) and Western Area having the lowest (275 [6·8%] of 4062). The Western Area (which includes the urban population in the capital city, Freetown) generally had significantly less frequent reporting of unsatisfied needs and significantly more frequent reporting of satisfied needs than the other three provinces.

**Figure 4:**
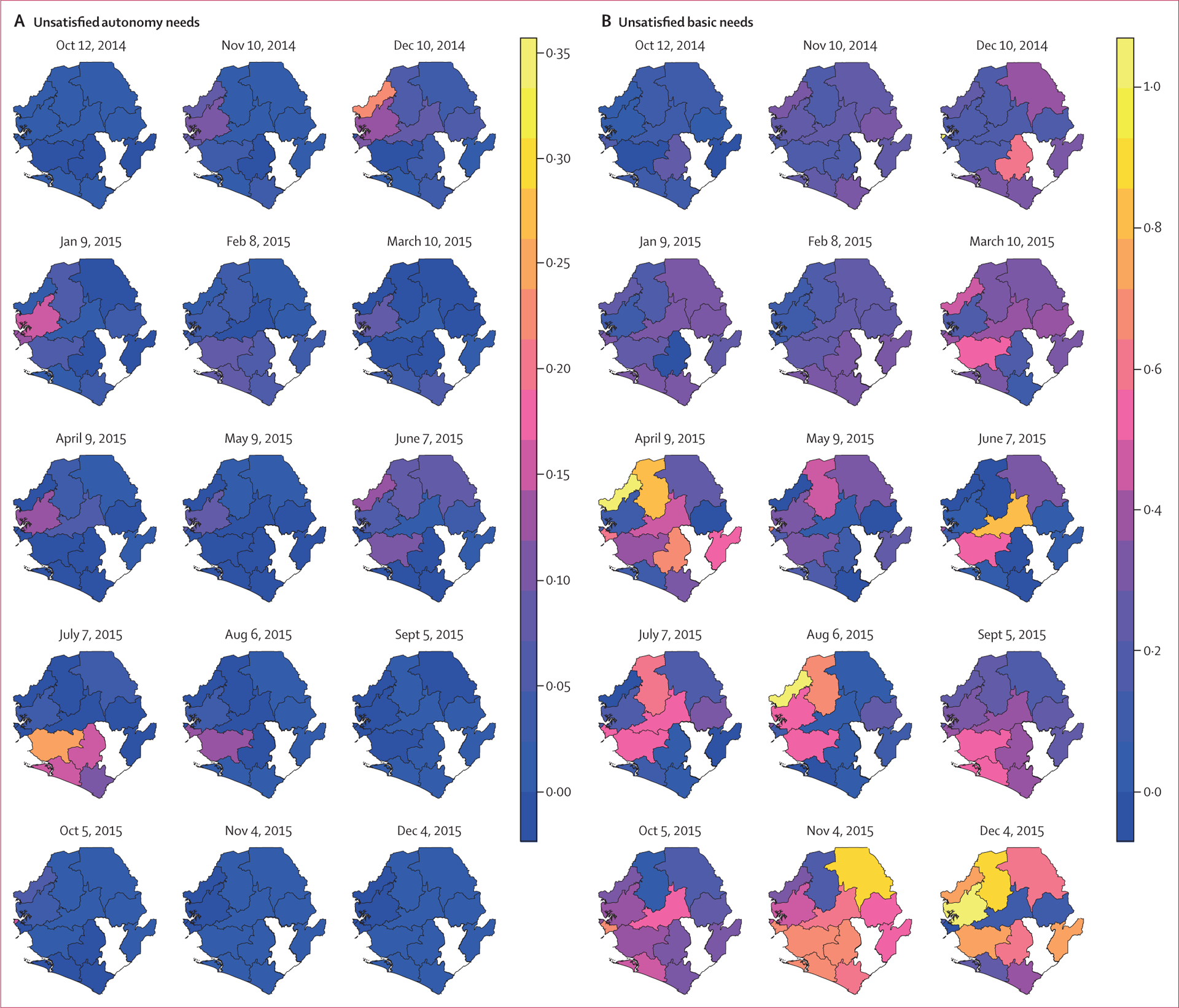

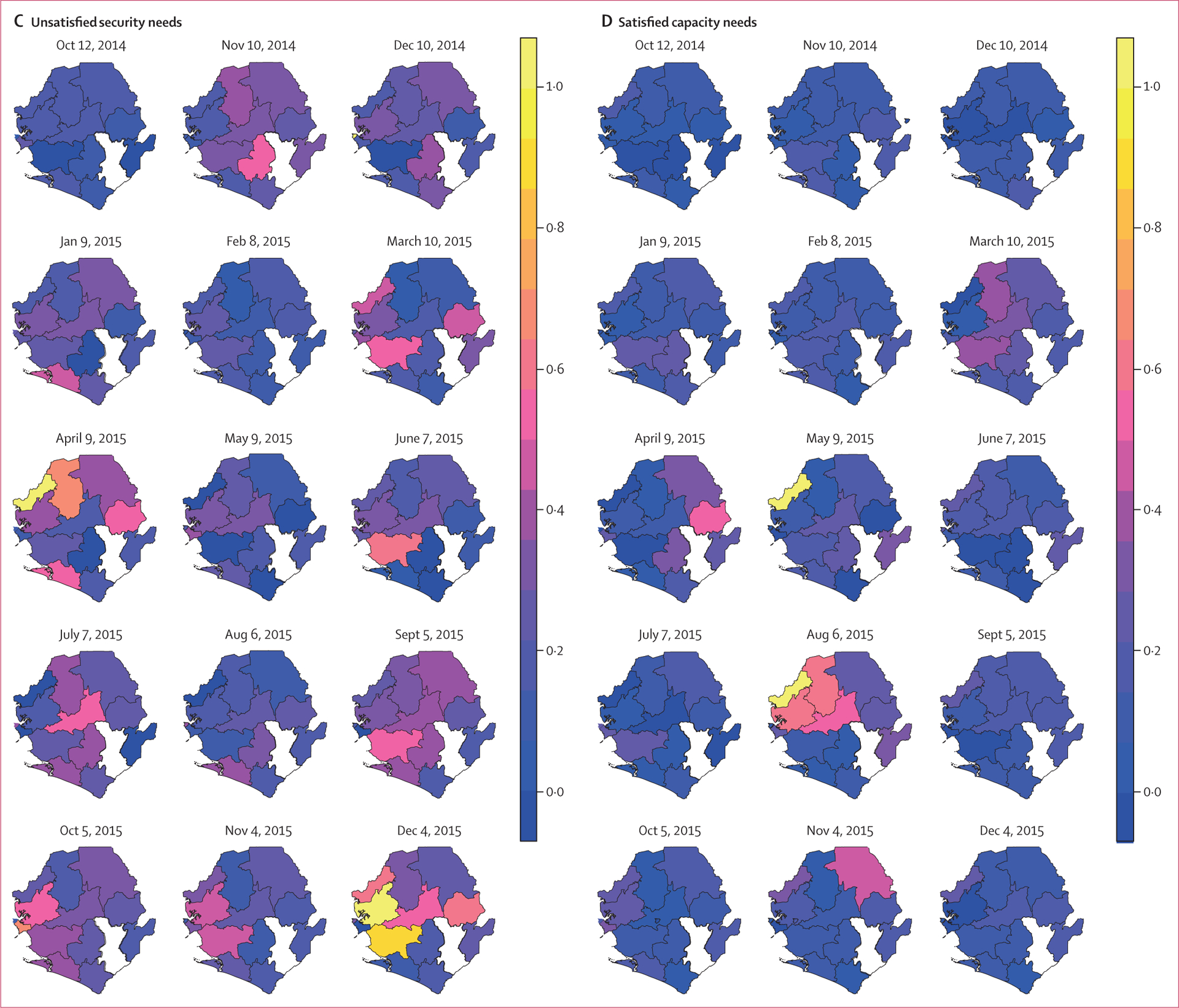
Spatiotemporal trends in district-level prevalence of needs category expression Sample maps are presented for unsatisfied autonomy needs, unsatisfied basic needs, unsatisfied security needs, and satisfied capacity needs. The maps represent once-monthly cross sections of the prevalence of a needs category after spatiotemporal interpolation. Prevalence was defined as the proportion of communities expressing a particular needs category divided by the total number of communities per space-time unit. Kenema was removed because of the limited data available in that district.

District-level temporal trends in the prevalence of unsatisfied and satisfied needs showed correlation with Ebola-related policies, campaigns, and shifting incidence (figure 4). Unsatisfied basic and security needs became increasingly prevalent across the country in March, 2015, which could be associated with hardship and fear due to increased transmission in February, 2015—resulting in the quarantining of about 700 homes and the reinstatement of previously lifted bans. In March, 2015, the British military presence (which was trusted on the whole) was withdrawn. This action reflected the fact that cases had dropped but was almost immediately followed by a case spike. Kambia and Bombali, where incidence was resurging in March, 2015, were expressing unsatisfied basic and security needs most prominently at that time. Furthermore, unsatisfied basic needs became highly prevalent again across the country as the epidemic waned, suggesting their endemicity in Sierra Leone. In April, 2015, districts with communities expressing unsatisfied autonomy corresponded with a reinforced health emergency in the north and west. Satisfied capacity needs in that same region a few months later could be reflective of resources from Operation Northern Push to address the renewed emergency.

**Figure 5:**
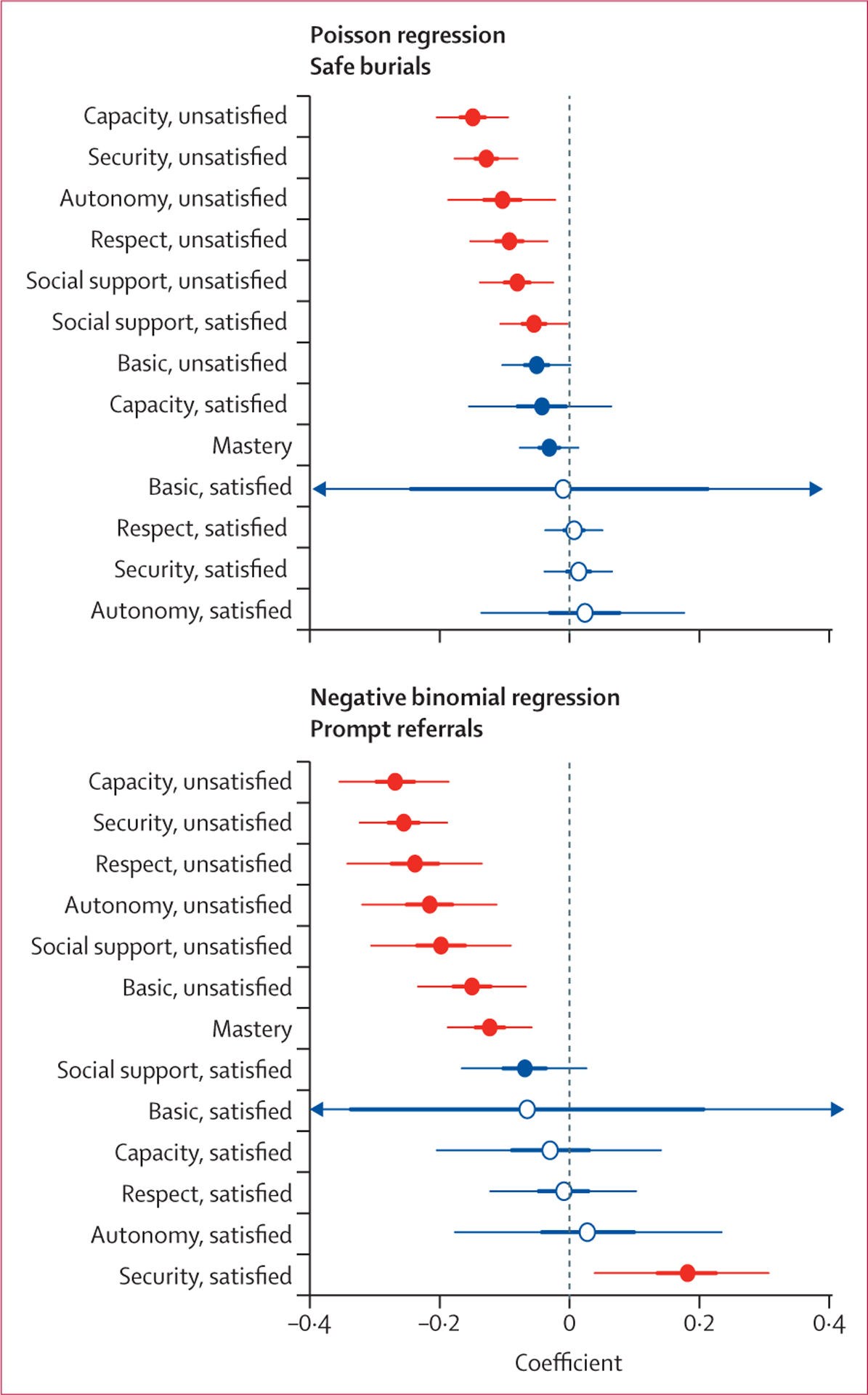
Posterior distributions for coefficients of Poisson regression of counts of safe burials and negative binomial regression of counts of prompt referrals to treatment within 24 h of symptom onset Thin and thick lines represent 95% and 50% credible intervals (CrIs), respectively. Parameters where 95% CrIs do not overlap 0 are indicated in red. Parameters where 95% or 50% CrIs overlap 0 are indicated in blue with closed or open circles, respectively. The CrIs around the coefficients for satisfied basic needs extend beyond the presented range for both outcomes.

In general, significant associations were observed for unsatisfied needs categories and the numbers of individuals referred to treatment within 24 h of symptom onset or buried safely (figure 5, appendix pp 17–18). Satisfied needs categories tended not to be significantly associated with behaviours, although the direction of their effects was generally null or positive. Coefficients for district-level effects were relatively small compared with those for needs categories.

Communities with unsatisfied capacity needs reported fewer safe burials (RR 0·86, 95% CrI 0·82–0·91) and fewer prompt referrals to treatment within 24 h of symptom onset (RR 0·76, 0·70–0·83) than did those who did not express unsatisfied capacity needs. The expression of unsatisfied security needs was associated with fewer safe burials (RR 0·88, 95% CrI 0·84–0·92) and fewer prompt referrals to treatment (RR 0·77, 0·72–0·83) compared with no expression of unsatisfied security needs. Unsatisfied autonomy, social support, or respect needs had similar although slightly attenuated effects and were associated with lower numbers of safe burials and prompt referrals (figure 5). Compared with community visits without expression of unsatisfied basic needs, those with concerns about basic needs had reports of significantly fewer prompt referrals (RR 0·86, 95% CrI 0·79–0·93) but no significant difference in the number of safe burials (RR 0·95, 0·90–1·00).

Associations between CLEA visit type (triggering *vs* follow-up visit) and the prevalence of satisfied versus unsatisfied needs were observed (appendix pp 7, 9, 18). Specifically, increased odds that a report came from a follow-up visit were significantly associated with expression of mastery (OR 1·48, 95% CrI 1·32–1·65), satisfied autonomy (OR 2·85, 2·04–3·88), satisfied capacity (OR 3·48, 2·70–4·42), and satisfied basic needs (OR 156·89, 1·65–310·39). All unsatisfied needs categories were associated with significantly decreased odds of a report being from a follow-up visit versus a triggering visit. As would be expected given the results relating unmet needs categories and response behaviour, follow-up visits were associated with significantly increased behaviour adoption. Relative to reports at triggering, follow-up visits were associated with higher numbers of prompt referrals to treatment (RR 1·40, 95% CrI 1·30–1·50) and safe burials (RR 1·08, 95% CrI 1·02–1·14), after controlling for epidemic week and accounting for district-level random effects.

## Discussion

Our findings quantify an association between unsatisfied needs and less practice of Ebola-safe behaviours at the community level during the 2014–15 response in Sierra Leone. Concerns about whether or not a community had autonomy, security, social support, respect, capacity to implement interventions, and basic needs were prevalent across the country and persisted over the duration of CLEA data collection. This finding demonstrates that communities affected by Ebola outbreaks have competing concerns that might be influencing collective motivation to undertake transmission-reducing strategies, such as prompt referrals to treatment and safe burials. Researchers and responders can understand the disconnect between response activities and the reactions of the population if there is an understanding of the underlying mechanisms associated with behaviour motivation.

Although existing theories of motivation provide insight into how needs influenced local behaviour during the west African Ebola outbreak, it is important to recognise that such decisions were occurring in an emergency-specific context of national-level and international-level decisions that generated and ultimately could address such concerns, particularly through the mediating influence of community engagement and social mobilisation. The CLEA approach^13^ used by SMAC involved triggering communities to act. By the end of the triggering visit, a community would have developed its action plan with specific by-laws for immediate implementation. The use of community meetings and engagement of local individuals to champion the action plans facilitated collective buy-in. The programme structure was intended to support communities to identify priority actions and act on these quickly, with the expectation that this would affect behavioural outcomes. The theory of change behind CLEA recognised triggering as the initiation of a feedback loop that included continued visits and participation in a responsive report system. This feedback approach is drawn from Participatory Learning and Action and Participatory Rural Appraisal^21^ used in HIV/AIDS interventions and Community-Led Total Sanitation programmes.^22^

The emergence of Ebola in a previously unaffected setting required the widespread introduction of new locally adapted sociocultural, health, and community engagement practices to control disease spread. Through the CLEA programme, communities developed action plans at triggering events and reported corresponding behaviour change during regular interaction with community mobilisers. While the two behavioural outcomes examined in this study were focused on reducing transmission directly and were uniformly measured across all engaged communities, the action plans^12^ included by-laws on a range of additional activities—eg, frequent handwashing, improved communication with ambulance and Safe and Dignified Medical Burial Teams, monitoring of visitors, and encouraging local donations of buckets and soap. Through these activities, communities would be addressing needs. For instance, unsatisfied security needs, such as fear of the response (eg, chlorine spray, speeding ambulance drivers), could be mitigated by action items including open dialogue with burial and ambulance teams to express the fear and request changes in how the chlorine was sprayed and in ambulance driving practices.

The impact of structured community mobiliser engagement that involved the development and monitoring of action plans in shifting communities towards empowerment warrants further analysis. As preliminary, motivating evidence, our findings demonstrate a trend towards more needs satisfaction following the peak intervention period and show that follow-up visits tended to be associated with the expression of satisfied needs categories, whereas triggering visits were associated with expression of unsatisfied needs. Furthermore, relative to triggering visits, significantly more prompt referrals and safe burials were observed during follow-up visits, thus suggesting that engaging communities might have inspired action with the potential to address unmet needs and reduce Ebola transmission. Notably, the roll-out of CLEA occurred at the same time that committed aid had reached Sierra Leone and facilitated action in communities that now had been engaged. For instance, the community care centres could accept increasing numbers of referrals and response services such as safe and dignified burial teams, ambulances, and surveillance were available. Needs expression was very likely sensitive to resources from other response programmes, in addition to CLEA-specific efforts. For instance, the expression of capacity needs satisfaction in the Northern Province and Western Province coincided with the roll-out of resources under Operation Northern Push. The overall expression of greater needs satisfaction by communities in the Freetown/Western Area could likewise demonstrate the urban-rural divide in a country with highly centralised health and economic structures,^23^ and thus geographical heterogeneity in resource availability for facilitating needs satisfaction.

Unsatisfied basic needs and needs related to present and future security were more consistently expressed throughout the epidemic than were unsatisfied needs related to social support, respect, and autonomy. We expect that pre-existing concerns associated with weaknesses in social and economic systems in Sierra Leone were exaggerated by the outbreak, leading to fears about future security (eg, teenage pregnancy, income) and current survival (eg, food shortages) that had limited short-term solutions. We contend that the process of triggering and structured community action planning should result in delineation of actions that a community knows it can adopt and sustain given constraints, while imposed actions can result in adoption and then abandonment. Under CLEA, many action plans included, among others, by-laws related to interactions with visitors, use of locally available materials for improved sanitation, and ways to better relationships with response workers, that were independent of external aid and most likely addressed needs for autonomy, capacity, mastery, social support, and respect.^12^ These low-cost or no-cost by-laws could be practically addressed by communities in Sierra Leone. We note, however, that such actions were addressing needs specifically imposed by the outbreak and adapting to the response.

Monitoring needs offers feedback on these realities and can guide community engagement efforts. We therefore advocate that such information is collected prospectively in ongoing and future Ebola outbreaks. The retrospective nature of this study required post-hoc mapping of community concerns to defined needs categories. However, our use of SVM to classify the data (through a supervised algorithm) and then to examine whether the data naturally clustered in a way resembling the needs categories (through an unsupervised algorithm) demonstrates the potential application of modelling to translate large-scale qualitative data into meaningful indicators of local action. Near-future work facilitating automated classification to provide real-time, evidence-based feedback to community mobilisers on how to further tailor their engagement strategies will be invaluable. We envision that community concerns digitally submitted on mobiliser reports would be automatically coded; results signalling communities at risk for limited acceptance of response behaviours could be sent as alerts to the responsible mobilisers. The results across communities could be used to generate maps that identify similarly at-risk regions where other response pillars would want to deploy resources, such as food. Implementation at this scale would warrant feedback on the sensitivity of different needs as indicators of response acceptance, which could change across settings, and the sensitivity of questions to elicit the most appropriate needs, as well as their level of satisfaction, accurately. For instance, our current findings support the negativity bias,^24^ or asymmetry in reporting that naturally tends towards the negative over the positive, in the expression of more unsatisfied versus satisfied needs. Questions structured to separately inquire about needs satisfaction and concerns about unsatisfied needs could be used. Likewise, distinctions between pre-existing and response-specific needs could be achieved through more precise framing.

It is important to note limitations to our study. We recognise that the recorded concerns might not reflect all needs within a given community at the time of reporting and that other community-level factors might have confounded the relation between needs and behaviour, such as proximity of health infrastructure, pre-existing economic status, and cohesiveness of social and governance structures. In addition, the reports were submitted by mobilisers, which might have created reporting bias because of poor memory or documentation of the community discussion. However, those concerns included were assumed to be prominently expressed to the mobilisers, of greatest concern to the community, and therefore most likely to influence behaviour. We note that role of desirability bias^25^ might have led to under-reporting or misreporting of numbers of sick people, deaths, referrals, and safe burials. The absence of other publicly available sources of community-level data on treatment-seeking and safe and dignified burials limits the ability to validate the outcomes used in the study. As a next-stage research effort, we will consider self-reported data alongside official epidemiological data from the Government of Sierra Leone, available through the Research Data Center of the National Center for Health Statistics. A recommendation for best practice by community engagement programmes during future outbreaks would be overlaying a verification system, such as through harmonisation and comparison between surveillance data and community-reported information in real time. This system would warrant strong integration across community engagement and other response pillars. For the present analysis, all visits were treated independently, irrespective of whether the same community was visited multiple times. Although geographical identifiers were recorded at the district, chiefdom, and community levels, incompleteness or inconsistent spelling in one or more fields prevented complete matching of follow-up and triggering visit datasets. Such limitations in data collected during the Ebola outbreak were common.^26^ Likewise, the collection of data for programmatic purposes led to inherent gaps in coverage, and thus information, in space or time because mobilisers were not uniformly deployed in all districts at all times.

Evidence-based guidance for community engagement social mobilisation efforts will have increasing relevance in the context of human behaviour change to address emerging and re-emerging infectious disease. To optimise their impact and reduce broad appropriation, community engagement programmes require study to understand the processes and principles that render them effective rather than just measures of their effectiveness.

The concepts of social mobilisation and community engagement have assumed different applications in outbreak response settings. A focus on risk communication and mass awareness-raising approaches may be done with contextually relevant messaging and can often focus on identifying and addressing rumour and attitudes to the response itself. However, although misinformation, rumours, and the questions about Ebola virus transmission were reflected in the data (capacity category), the interpretation of our results in the context of motivation theory and an overarching ecological framework suggests that actionable change will involve approaches that promote local ownership of the response based on community-defined actions that are protective while consistent with local interests. Interdisciplinary and participatory processes involving communities as well as health-care workers, epidemiologists, statisticians, and social scientists will be important to determine strategies for efficient control of widespread outbreaks by bridging of theory, observation, analysis, and intervention.^27,28^ Data collection on behaviour change with intentional framing of questions to solicit relevant needs information will be important for enhanced, real-time understanding of which environments are most supportive of motivating health behaviour and of how to tailor outbreak response strategies.

## Supplementary Material

Supplementary appendix

## Figures and Tables

**Table:** Needs classification scheme

	Definition for needs satisfaction*	Sample themes or terms†	Sample role of community engagement in needs satisfaction
Basic	Having food, water, and shelter, as well as the ability to access resources to satisfy basic needs	Food, drinking water, more poverty, higher prices, no trade or market	Mobilisers advocate for resource allocation to communities with expressed needs for food and water
Safety and security	Feeling safe in the present or secure about the future, including issues around national development and family stability	Fear because of ambulance speed, issues with chlorine spray, school closures, rise in teenage pregnancy	Community members can independently or with facilitation of the mobiliser request that ambulance drivers take more caution
Social support	Being able to depend on others, including trusting their intentions and counting on them in an emergency	Distrust, corruption, Ebola is man-made	Interaction with mobilisers provides communities with a trusted point person for questions and concerns about the response
Respect and pride	Feeling treated with respect or in a manner that was fair, irrespective of status (economically, physically, etc)	Manner of treatment at hospitals, disrespect for survivors, attitude of burial team	Communities are encouraged to express their concerns around the burial practices through open dialogue with response teams, leading to change in the practice at a local or policy level
Autonomy	Being able to choose how time is spent and freedom in decision making	Movement restricted, hunting not allowed, normal activities on hold	Communities decide upon and realise their own plans of action with supportive monitoring from mobilisers
Mastery	Having the experience of learning something new or accepting and adapting to a new situation	Ebola is real, reiteration of by-laws, widespread handwashing	The process of successfully undertaking a community-level plan of action demonstrates acceptance and adaptation, such as by refusing visitors into the communities or locally enforcing no handshake policies
Capacity	Having the information, skills, and equipment to execute desired action	Buckets, chlorine, training, sensitisation	Communities rally collective action, such as donation of soap or buckets to fill capacity gaps; mobilisers advocate for training for communities with expressed needs

* Adapted from classification scheme proposed by Tay and Diener.^14^

† All themes classified per needs category are included in the appendix (pp 10–14).

## Research in context

### Evidence before this study

We searched PubMed for original studies on community engagement and social mobilisation programmes promoting behaviour change related to infectious disease. Articles published from database inception to May 1, 2019, in any language, were considered. Search terms included (“community engagement” OR “community mobilization” OR “social mobilization”) AND (“behavior change” OR “prevention”) AND (“infectious disease” OR “Ebola” OR “outbreak” OR “public health emergency”). Among the 89 search results, we identified 36 abstracts for publications that were not outbreak reports, commentaries, or review articles; were focused on infectious disease; and made explicit reference to social mobilisation or community engagement programmes beyond their use as a methodological approach, such as for participant accrual. 19 of the 36 results were not evaluations of community engagement or social mobilisation efforts, but rather recommended such programmes on the basis of their results. An additional 11 articles reported on descriptive qualitative studies or process evaluations with no or limited focus on behavioural outcomes associated with community engagement. Finally, six studies were intervention-based or quantitative survey studies that assessed the role of community engagement in encouraging preventive behaviour against Ebola, polio, schistosomiasis, HIV, and general infectious diseases in a post-earthquake setting. Existing, largely qualitative evidence supports an ecological framework for the role of community engagement and social mobilisation in outbreak settings with multiscale response actors and policies. To our knowledge, no studies have explored the role of community engagement in mediating the influence of outbreak setting needs on local behaviour change.

### Added value of this study

We provide an ecological framework for the role of community engagement in community-level needs satisfaction through both resource allocation and promotion of locally developed and implemented action plans. Adding to the extensive, largely qualitative evidence that describes facilitators and barriers to behaviour change during the Ebola outbreak, our framework uniquely draws on theories of motivation to understand local behaviour in settings with multiscale response actors and policies. Furthermore, our classification of large-scale qualitative data to provide statistical evidence for the role of diverse needs in local behaviour change offers actionable guidance for future outbreak response. We demonstrate how community-voiced concerns could serve as a measure for more effective engagement by community mobilisers. Specifically, we provide evidence to guide information collection and introduce preliminary methodology for automated classification of large-scale qualitative responses that could offer real-time feedback.

### Implications of all the available evidence

Community engagement and social mobilisation are widely recommended as fundamental to the success of behaviour-focused interventions against infectious disease. The influence of satisfying diverse needs—including basic physiological needs and needs for security, autonomy, respect, social support, and capacity—on the adoption of behaviour change has implications for such programmes. Prioritising community-based action plans ensures adoption of locally feasible, locally owned behaviour change through processes that satisfy needs, with the support of and resources allocated through community mobilisers; our results indicate that fewer unmet needs will lead to improved retention of preventive behaviour change during the course of an outbreak.
