# Supplementary material for: Unmet needs and behaviour during the Ebola response in Sierra Leone: a retrospective, mixed-methods analysis of community feedback from the Social Mobilization Action Consortium
Source: Lancet Planet Health. Author manuscript; Available in PMC 2021 Jun 6. (PMC8180180; doi:10.1016/S2542-5196(20)30008-5)
Supplement: Supplementary appendix [file NIHMS1708545-supplement-Supplementary_appendix.pdf]

THE LANCET
Planetary Health

Supplementary appendix

This appendix formed part of the original submission and has been peer reviewed. We post it as supplied by the authors.

Supplement to: Skrip LA, Bedson J, Abramowitz S, et al. Unmet needs and behaviour during the Ebola response in Sierra Leone: a retrospective, mixed-methods analysis of community feedback from the Social Mobilization Action Consortium. *Lancet Planet Health* 2019: **3:** e74–85.

Supplementary Material

Methods

Statistical Model

Suppose that i denotes the community and j denotes a situation warranting intervention, with $j = 1$ referring to reports of seriously sick individuals in the community and $j = 2$ referring to reports of deaths in the community. Let y_{ij} represent the number of seriously sick individuals referred to treatment within 24 hours ($j = 1$) or the number of deaths responded to with safe burials ($j = 2$) in community i. Let x'_{in_q} be a vector of n covariates representing categories of needs. Note that every n^{th} covariate is expressed as either gains (subscript 1) or losses (subscript 2) in the indicated needs category. Separate vectors of coefficients, $\boldsymbol{\beta}$ and $\boldsymbol{\beta\lambda}$ were considered to evaluate differential effects of unsatisfied versus satisfied needs categories, respectively. Then, a Poisson model describing y_{ij} is given by

$$y_{ij} \sim Poisson(\mu_i) \;\; for$$

$$log(\mu_i) = D_i + \boldsymbol{\beta}\left(x'_{in_1} + \boldsymbol{\lambda} x'_{in_2}\right) + \beta_M \mathrm{M} + log(m_{ij}) \;\; and \; D_i \sim \Gamma(\alpha, \sigma)$$

where M is the binary term for expression of mastery, the offset term m_{ij} is the number of seriously sick ($j = 1$) or the number of deaths ($j = 2$) reported by the jth community, and α and σ are hyperparameters for the random effects term accounting for the district D of community i. Similarly, the negative binomial model would be given by

$$y_{ij} \sim NegBin(p_i, r) \;\; for$$

$$p_i = {}^{r}\!/_{(r + \mu_i)} \;\; and$$

$$log(\mu_i) = D_i + \boldsymbol{\beta}\left(x'_{in_1} + \boldsymbol{\lambda} x'_{in_2}\right) + \beta_M \mathrm{M} + log\left(m_{ij}\right) \; and$$

$$r \sim unif(1{,}30) \; and \; D_i \sim \Gamma(\alpha, \sigma)$$

Time and visit type (triggering versus follow-up) were not explicitly included in models with behavioural outcomes due to strong correlation of changes in needs expression with these effects. A separate analysis was conducted to establish the relationship between visit type and behavioral outcomes, after controlling for time. All covariates were treated as binary. This approach was expected to improve the interpretation of the results while focusing more on frequency with which a needs category was reported rather than the intensity of the need (*i.e.*, more or less intense if a single category versus multiple were reported). The approach is consistent with theories on the relative relevance of frequency and intensity on gauging self-reported sentiments. [19]

Fitting of regression models was implemented in JAGS using Markov chain Monte Carlo (MCMC) sampling. For each model, three chains of 100,000 model iterations were run following a burn-in period of 5,000 iterations. Model convergence was evaluated with the Gelman–Rubin statistics. Non-informative priors were assumed for all coefficients $\boldsymbol{\beta}$ and $\boldsymbol{\beta\lambda}$ of the unsatisfied and satisfied needs categories, respectively**.**

F1. Most Frequent Words Appearing in Categorized Responses. The 20 most frequent words, excluding “Ebola,” numbers, and stop words, are represented for each needs type to reflect consistency of classification with intended meaning of the category. The frequency reflects word counts across all responses (versus the number of responses containing each word).

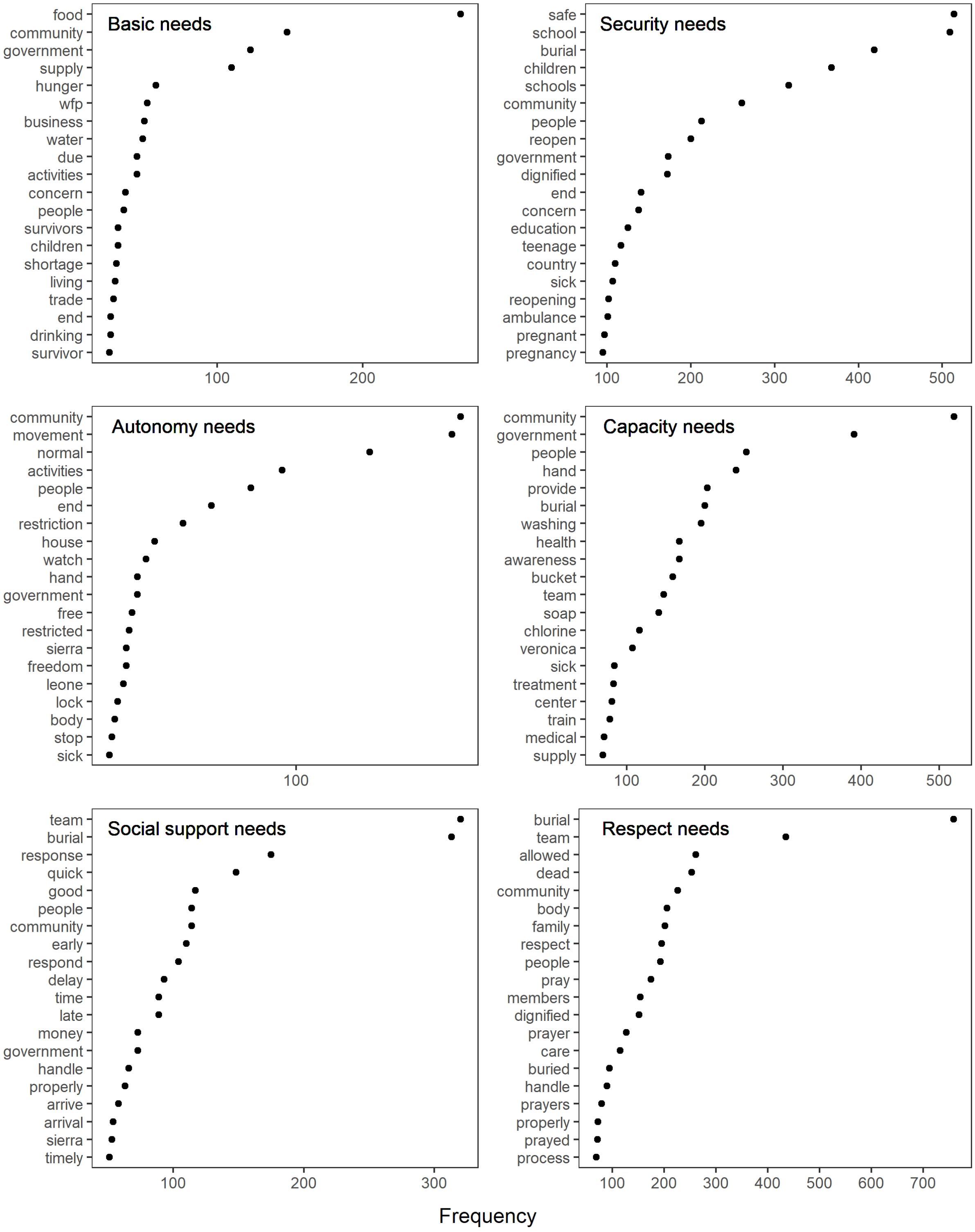

F2. The quality of best match between topics found by unsupervised clustering (Latent Dirichlet Allocation) and manually coded needs concerns. For each number of potential topics (i.e. N clusters) (x-axis), LDA was performed to produce that number of unique topic clusters. For each individual topic (in the N clusters) the marginal probability of a survey answer appearing in that topic cluster, given that that survey answer was manually coded as “Concern #6: Respect Needs” is computed. Of these N topics, the one with the highest marginal probability (y-axis), is plotted (in red). This best-fit topic, is the one that co-occurs most frequently with this need concern – i.e. survey responses label as “respect needs” tend to be clustered into this best-fit topic more often than into any other cluster. For example, with 8 topic clusters, around half of all survey responses label as Respect Needs are grouped into the same topic cluster (topic #2, according to Table 3), based only on the text in those open-ended survey responses. As a control, the topic with the largest probability (unconditional on any need concern) is also plotted (in blue). The improvement in predicting the topic that a survey response falls into, when it is known that they response was given a “respect need” label (gray area), demonstrates the mutual information between automated topic clusters and manual coded labels. This mutual information may provide evidence that the chosen need concern labels matching the naturally occurring themes in the content of the survey responses, that the manual labeling of responses to need concern classes is based on these themes from the content of the survey responses, and perhaps that in future work automated methods based on the content of responses (similar to our SVM method employed here) might provide some assistance or verification in need concern label coding.

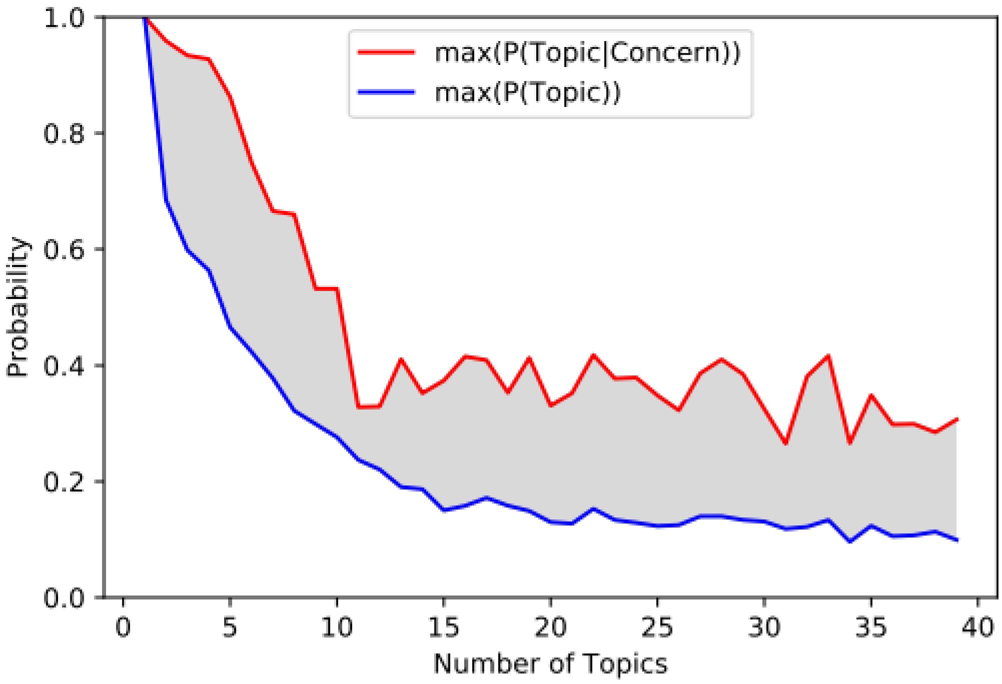

F3. The ability to predict the best-fitting topic label for survey response, given you know that this response was manually coded with a given need-concern label, plotted for all seven need-concern labels. Here, the baseline control curve (in blue) now corresponds to the average frequency of all topics, rather than the maximum topic frequency – which assumes that responses from all concern-label are mapped across all topics, rather than different concerns all mapping to the most frequent topic (as would be the case if the need-concern label held no mutual information with the topic label). This assumption is supported by the mapping shown in Table 3. Note that all concerns (1-7) always show marginal probabilities greater than baseline, suggesting that there is consistently mutual information between automatically extracted topics and all of the manual need-concern labels.

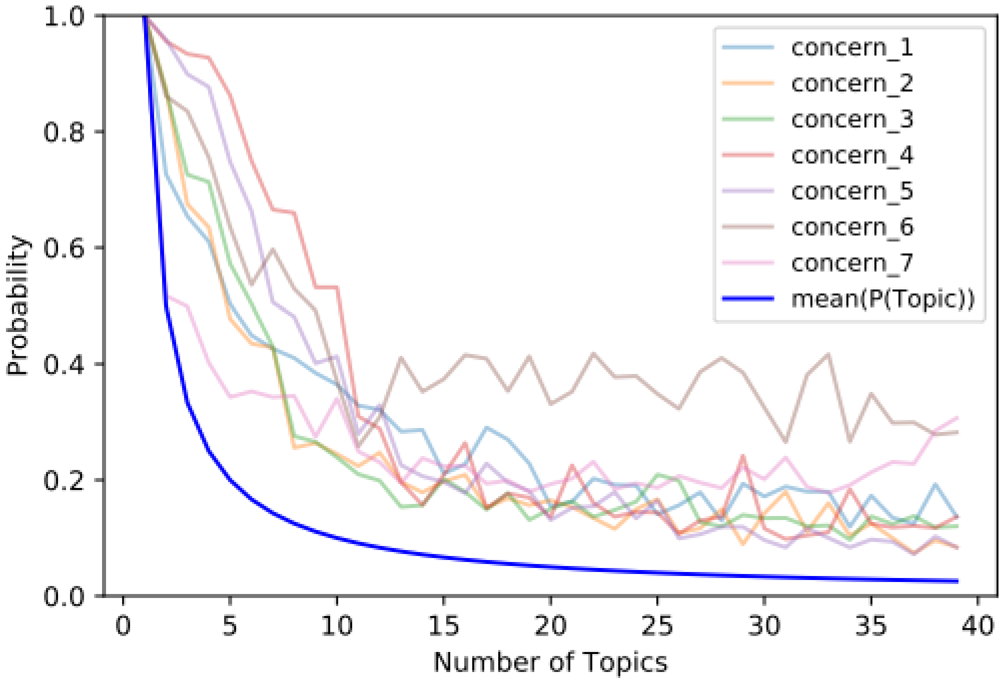

F4. The ability to predict the best-fit topic of a survey response given its need-concern label (*P(topic|concern),* in red) becomes easier as the number of topics increase and the content of each topic becomes more and more specific (resulting in a single word per topic, in the limit of the many topics). Conversely the probability of correctly predicting the need-concern classification of a survey response, given its topic (*P(concern|topic),* in blue) initially gets easier as more unique topics allows for greater discrimination, but then falls on average as additional topics are found that do not correspond closely to one of the seven need concerns (i.e. does not discriminate between need concerns better than the average topic in the other clusters). That the number of topics that leads to the highest average need concern predictability per topic ($argmax_{num\text{-}topics}$*(mean(P(concern|topic) for all topics)*) occurs at 8 automatically clustered topics, which also corresponds to the intersection of the *P(topic|concern)* curve and also is similar to the true number of needs-concerns classes, suggests the potential for need concerns and topics to both represent the major themes in these responses. The intersection point of these two curves, reveals the best tradeoff between the precision and recall of the topic to cluster mapping.

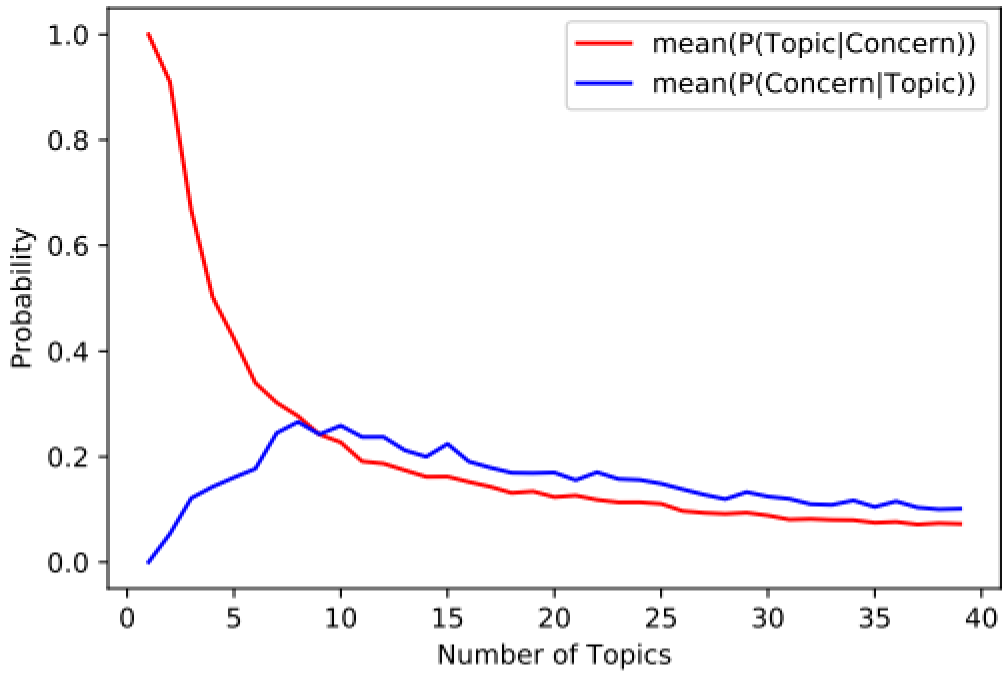

F5. The intersection point of the predictability of the single best-fit topic for the most effectively fit need-concern label (*max*$_{topic}$*(P(topic|concern #6))* and the ability to predict this concern by a given topic ((*max*$_{topic}$*(P(concern #6 |topic))*) occurs between 8-9 topics. This further contributes to the evidence that this number of topics balances recall and precision. Though by focusing only on the most relevant topic to a single need-concern (rather than the average performance of all topics, as in Fig. 7) the best fit topic (*max(P(concern|topic)*) continues to increase as the number of topics increases (further refining each topic), until over 80% of survey responses in this topic fall into the same need-concern class.

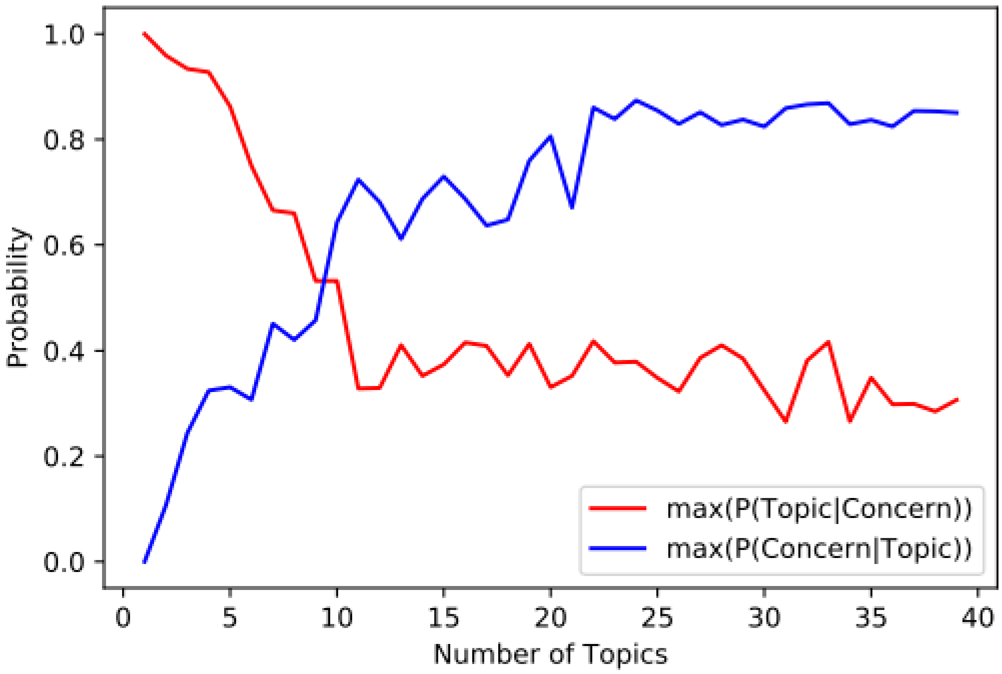

F6. Number of Communities Engaged per Week by Mobilizers of the Social Mobilization Action Consortium (SMAC)

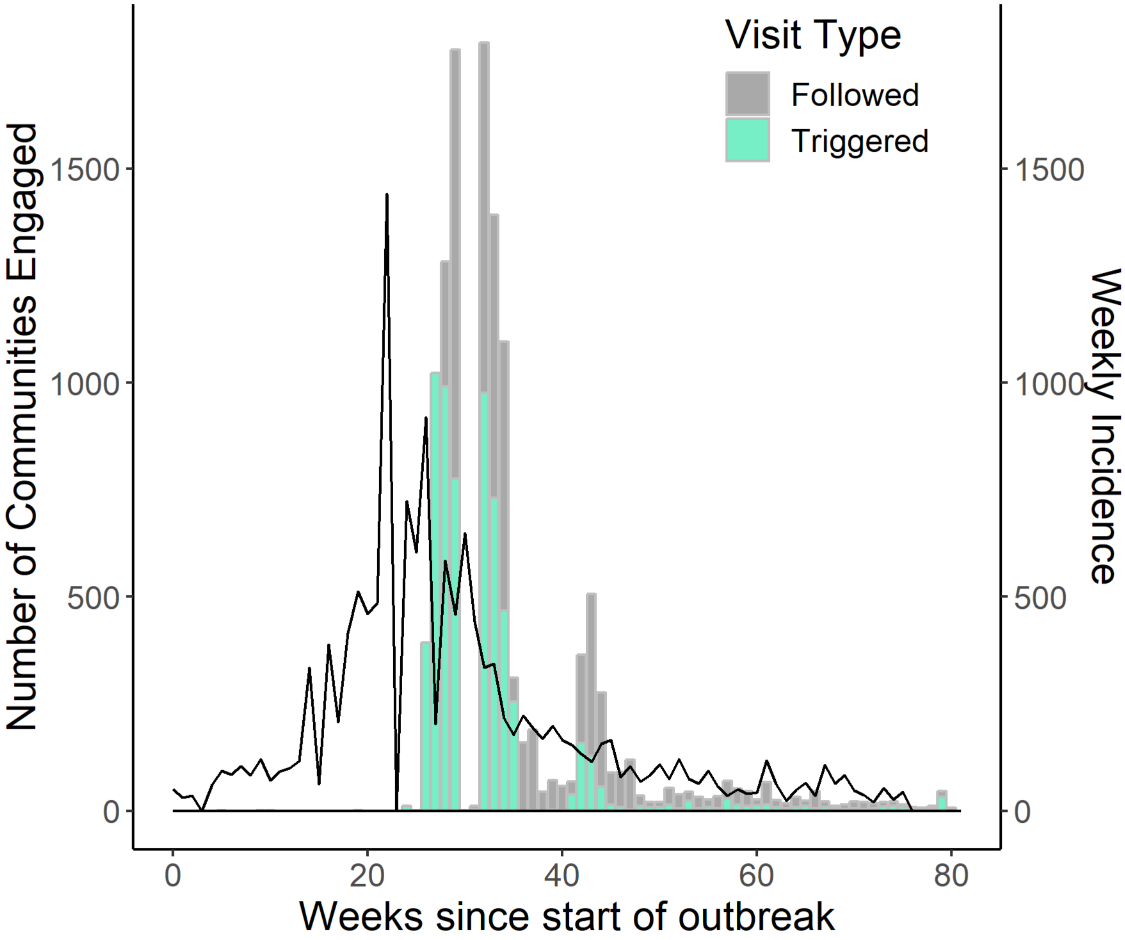

F7. Distribution of Needs Classifications by Province of Sierra Leone. Each ring represents the proportion of community visits during which a particular needs category was expressed. Red rings reflect unsatisfied or unmet needs categories, while blue reflect satisfied or met needs categories.

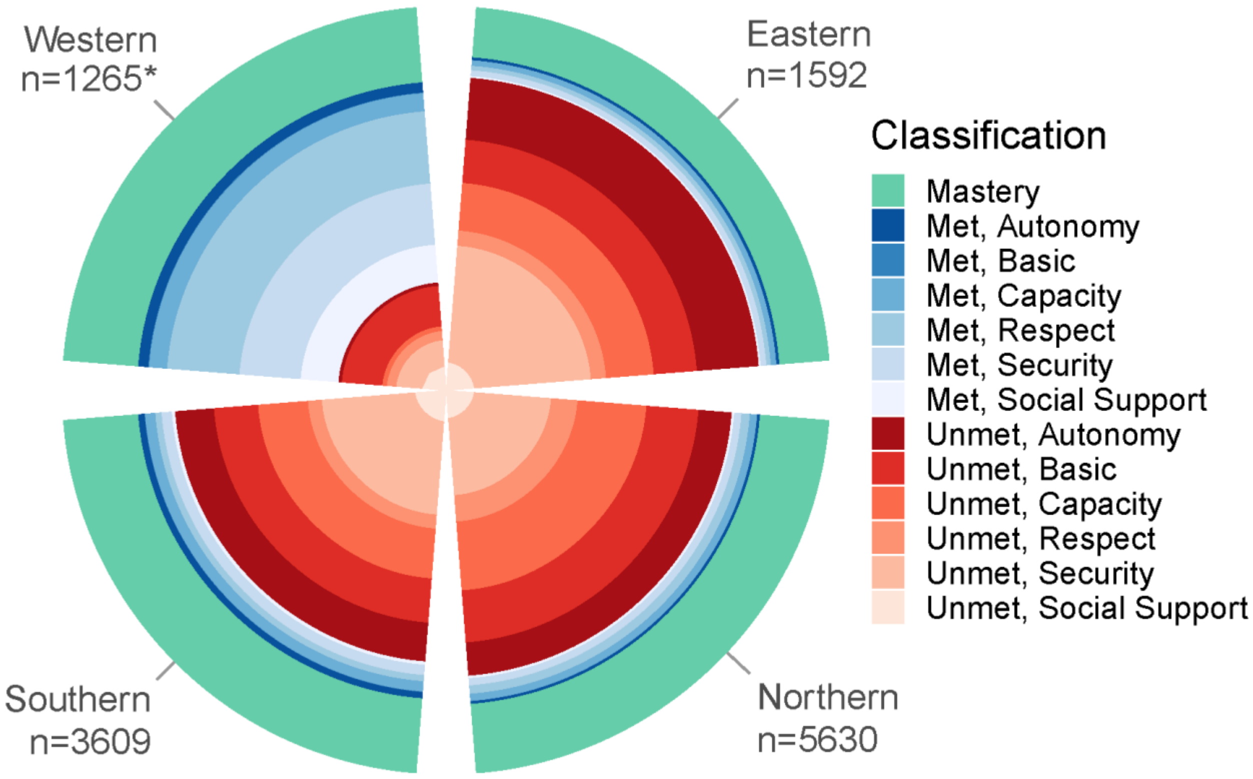

* Number of visits per province

F8. Posterior Distributions for Coefficients of Logistic Regression on Relationship between Needs Categories and Follow-Up versus Triggering (Reference) Program Status

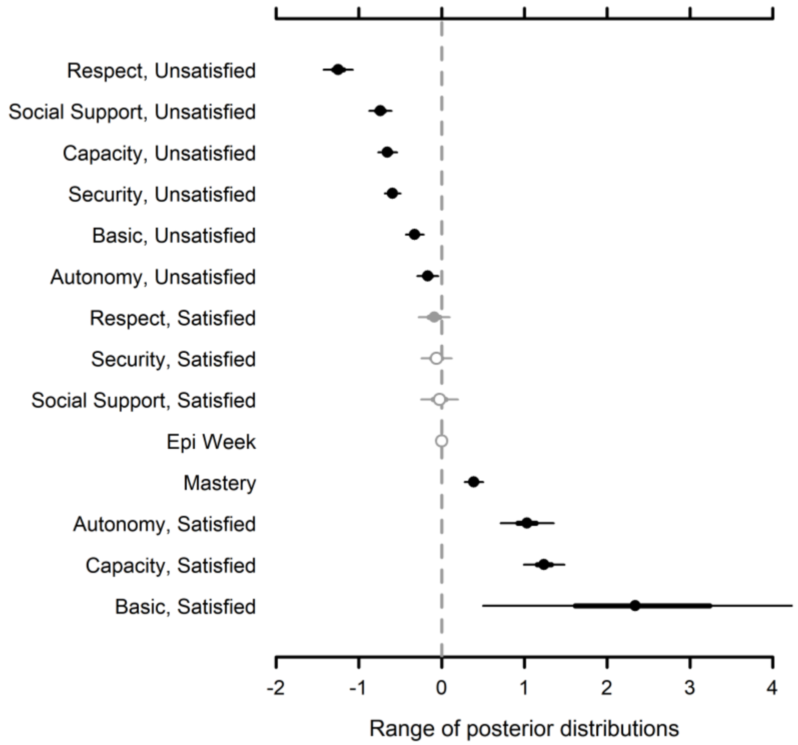

* Thin and thick lines represent 95% and 50% credible intervals, respectively. The credible intervals around the coefficient for satisfied basic needs extends beyond the presented range to the right.

T1. Themes to Needs Classification

	Theme/Description of Concern	Frequency[1]
Unmet, Basic Needs	No food (shortages in community, market closed)	414
	No trade/business/government jobs	371
	Community has not received food supply from aid groups	172
	Economic crisis/hardship including joblessness	156
	No support for Ebola-affected individuals	143
	Higher prices of food and/or transport	100
	No clean drinking water	92
	No food for quarantined individuals/households	71
	No farming/agriculture/harvesting and/or farm land destroyed	71
	More poverty in community	35
	Food supply eaten by animals or otherwise spoiled	9
	No food for survivors and sick	6
	Fewer commodities available	4
	Inadequate dress/clothing	1
Unmet, Safety/Security Needs	Schools need to open, children are sitting idly	1240
	Increasing frequency of teenage pregnancy/teenage marriage	290
	Intervention spray is harmful	184
	We are "going backwards"; illiteracy; development on hold	156
	Fear of Ebola (Worse than rebel crisis, so much death, no boundaries)	147
	Fear/Alarm due to response practices (PPE, Ambulance Alarm, Ambulance Not Safe)	141
	Lawlessness and lack of enforcement, noncompliance with bylaws/disbelief	132
	Separate place for survivors (holding center for 3 months)	64
	Symptoms of Ebola are similar to other diseases that existed before	57
	Concern over continuation of interventions (Bushmeat) post Ebola	46
	Need for compensation post Ebola (Free education, money for survivors, free healthcare, permanent treatment centers)	43
	Fear of Hospital	40
	Digging of shallow graves by burial teams	39
	Poor roads inhibiting response	34
	Need for strangers to stay out	32
	Malaria tablets have side effects	29
	Uncertainty about Future	29
	Increasing/rapid spread of virus	24
	Should be separation between Ebola and non-Ebola patients	23
	All deceased are buried by burial team even if not sick with Ebola	21
	PPE and gloves scattered in community	20
	No security for or monitoring of quarantined homes	19
	Shared toilets and washing facilities causing disease	17

[1] Frequencies with which concern appeared; multiple concerns were often communicated and coded within or across categories

	Concern about post-Ebola economic status	17
	Fear of survivors; survivors not welcome in community	16
	Need for safety at school/fear over reopening of school	14
	Ebola has affected us seriously	13
	Post-Ebola control of animals/bushmeat to prevent recurrence	12
	Fear of people not returning after being taken to ETU	11
	Need for Ebola to stay out of village	10
	Fear/insecurity due to mass gathering for food supply	9
	Burial teams have been involving community members without adequate protection	8
	Need for safety in terms of restricted movement	8
	Afraid to call 117	7
	Need for free education	7
	Ebola worse than or similar to conflict	6
	Survivors should be on burial teams to prevent more sickness	5
	Will we return to normal burial practices after Ebola?	5
	No cure	4
	People from holding center wandering around community	3
	More security for checkpoints/schools needed	3
	Maintain treatment centers after Ebola	3
	Sick outsiders are seeking care in their community	2
	Abuse of children	2
	Condition of the Country	2
	Ongoing sensitization and reiteration of bylaws necessary post Ebola	2
**Unmet, Autonomy Needs**	Movement Restricted	713
	Overall/Normal activities on hold	188
	No traditional entertainment/No secret societies or public gatherings	149
	No handshaking/less body contact allowed	103
	Practice of eating animals in town changed	56
	Lack of Freedom	20
	Change in their caring for the sick	20
	Not happy with government bylaws and imposition by outsiders	17
	No travel to Mecca	11
	No cultural practices allowed	4
	Need for reduced bylaws since cases are decreasing	4
	Use of the Military to restrict	3
	No hunting allowed	2
	Pregnant women have to deliver elsewhere (not in community)	2
**Unmet, Capacity Needs**	Materials needed to execute the response (Gloves, thermometers, buckets, soap, chlorine, ambulance)	804
	Using local community members on burial teams	241
	Need for community health facility/improved healthcare	228
	Need for training (Burial Teams, Youth, First Aid)	150
	Need for knowledge (What to do with the sick, IPC, sensitization, how did Ebola start?)	102
	Information about the Ebola vaccine, including why it is delayed	87
	Need for medication/vaccine/doctors	70

	Need Ebola medication/treatment (for suspected cases)	66
	Need for sensitization campaigns and community-level education	58
	Information on signs and symptoms and transmission of Ebola	51
	Misinformation or no information; questions about Transmission, how there are survivors if no cure exists, how burial team is not sick	50
	Need for malaria treatment	37
	Need for ORS	32
	Support for task force needed	29
	Long distance to nearest health facility for care	29
	Need for free healthcare to address all illnesses	26
	Need treatment for all sicknesses	23
	How to prevent Ebola?	21
	No toilets and sanitation infrastructure	21
	Testing and treatment needed for people in quarantine	21
	Better distribution of doctors (from abroad)	19
	Need for information on transmission and disease: Is Ebola spread through handshake? Why are doctors dying of Ebola? Why do some survive and others do not? Why is Ebola so deadly? Is Ebola really preventable?	16
	Need for community members to make up the response teams (contact tracers, surveillance, burial teams, etc.)	14
	Need for information on caring for the sick	11
	Information needed on how to interact with and treat survivors	9
	Hand washing difficult to observe	8
	Poor access to health facility	8
	Need for contact tracers from the community	5
	Need for local ERT	2
	Radio education not effective for rural areas	1
**Unmet, Social Support Needs**	Cannot rely on Burial Team/Hotline Response/Ambulance; delayed response	434
	Distrust in others/Separation in the Country	103
	Need for others to join their collective effort	76
	Corruption	66
	Outsiders brought Ebola for money or other reasons	48
	Loved ones separated/interactions affected	47
	HCWs do not want Ebola to end due to the salaries they are getting	43
	Death of family members/loved ones	31
	Only poor people are suffering from Ebola; rich people are not complaining of Ebola	26
	No communication by response team after taking community members	18
	Ebola is manmade	15
	Ebola is a curse	10
	No communication on cause of death after testing	10
	Poisoning of water/distrust in government	9
	Children now defying their parents	4
	Is Ebola real?	2
	Need for communication with loved ones in other districts	1
**U n**	Manner of burial by burial team, even when not Ebola	296

	No respect for burial traditions/bad attitude of burial team toward community	161
	Community members/family/religious leaders restricted from active engagement in burials	96
	Respect/better treatment at health facility or by burial team members	86
	Want their loved ones to be buried in community/To see them even if not to touch them	32
	Neglect of pregnant women due to fear of Ebola	26
	No benefit to community for participating in response	16
	Banning of traditional healers/No involvement of traditional leaders	10
	Disrespect of survivors	7
	Lack of follow up on/contact with patients taken from the community	4
	Unfair treatment of quarantined homes	3
	Same burial irrespective of socioeconomic status	2
Mastery	Acceptance - Ebola is Real	521
	Practicing regular handwashing	412
	Acceptance of bylaws and government rules	380
	Acceptance of referral to treatment/calling 117	303
	Acceptance of the burial team and SDB practices/gain of chiefdom-level burial teams	280
	No case or death recorded	217
	Maintaining good sanitation, a clean community	134
	Happy with response and Ebola coming closer to an end	103
	Reiteration of bylaws in community	88
	No accommodation of strangers	79
	No concerns	56
	Reporting of sickness/deaths/no secret burials	55
	No public gathering in accordance with by laws	36
	Acceptance of no eating bushmeat	25
	Acceptance - Ebola is a Killer Disease	24
	Confirming implementation of safe and dignified burials	15
	Early treatment can lead to survival	15
	Acceptance of no public gatherings	15
	Acceptance of restricted movement	9
	At least partial (majority) adherence to the by laws	7
	Ebola can be prevented	3
	Happy with the current practice of the burial team	3
Met, Basic Needs	Benefiting from food supply	9
	Business activities have restarted	4
	Have access to good water supply	1
Met, Safety/Security Needs	Less fear of Ebola/gain in safety	800
	Handled with care	97
	Schools are open	45
	No fear of hospital	12
	Acceptance of Ebola survivors into community	8
	Fewer sick and dead	6
	No fear due to ambulance alarm	5

	No more fear of the burial team	4
	Better roads/bridges constructed	2
	Less teenage pregnancy	1
Met, Autonomy Needs	Committee set up (to enforce bylaws)/mounting of checkpoint/taskforce/surveillance	155
	Community is taking the lead	41
	Movement has been restored	22
	Active participation by women in the community	9
	Public gathering now allowed in community	5
	Started football and other cultural activities/public gatherings	5
	Return to normal activities	2
Met, Capacity Needs	Gain in sensitization/awareness	348
	Increased care seeking possible	49
	Gains of supplies (buckets, soap, etc.) from community members or outside sources	43
	Local holding center or health facility built	18
	Center for quarantining people was created	5
	Vaccines being administered	2
Met, Social Support Needs	Happy with medical teams/quick response/best people to do job	997
	Good cooperation and concern for one another	96
	Improved relationships with health workers (such as through community talk)	13
	Social support from HCWs	8
	Interactions with loved ones/family restored	3
	Confidence in burial team	2
	Improved relationships between community members; no more fighting	1
	Gain in support for survivors	1
Met, Respect Needs	Involvement of family in burial process/allowed to pray	935
	Burials are now safe and dignified	353
	Respectful burial team	296
	Better treatment in health facilities/respect for the sick or dead	106
	Pregnant women seeking care/ANC	9

T2. Mapping of each concern to its best matching topic (i.e. maximizes P(Topic|Concern)) for 8 topics.

Given Concern	*Most Relevant Topic*
1: Basic Needs	Topic #1
2: Security Needs	Topic #4
3: Autonomy Needs	Topic #7
4: Capacity Needs	Topic #8
5: Social Support Needs	Topic #3
6: Respect Needs	Topic #2
7: Mastery	Topic #5

T3. Mapping of each topic to its best matching concern (i.e. maximizes P(Concern|Topic)) for 8 topics.

Given Topic	*Most Relevant Concern*
Topic #1	1: Basic Needs
Topic #2	6: Respect Needs
Topic #3	5: Social Support Needs
Topic #4	6: Respect Needs
Topic #5	7: Mastery
Topic #6	4: Capacity Needs
Topic #7	3: Autonomy Needs
Topic #8	4: Capacity Needs

T4. Word cloud (most frequent words, sized by frequency) for each automatically (unsupervised) topic cluster.

Topic #1

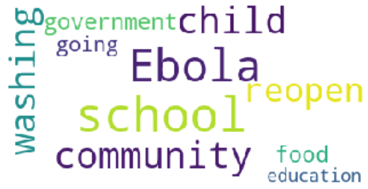

Topic #2

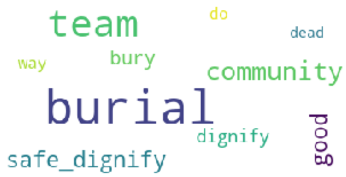

Topic #3

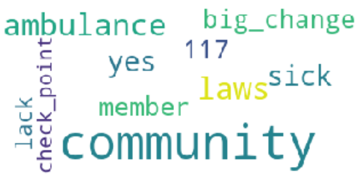

Topic #4

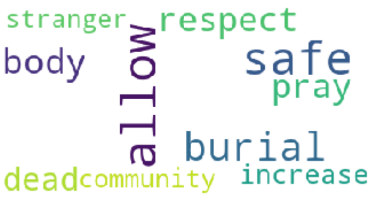

Topic #5

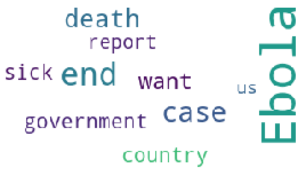

Topic #6

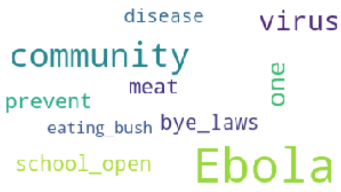

Topic #7

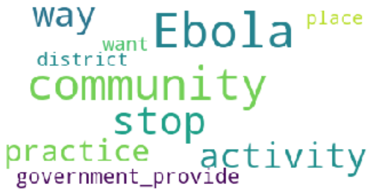

Topic #8

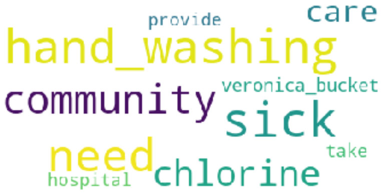

T5. Coefficients for Hierarchical Bayesian Regression Count Models of Behavioural Outcomes

**Covariable**	**Outcome**	
	Referrals to Treatment	Safe and Dignified Burials
Unsatisfied Basic Needs	-0.151 (-0.235, -0.068)	-0.050 (-0.103, 0.003)
Unsatisfied Security Needs	-0.256 (-0.323, -0.189)	-0.127 (-0.176, -0.078)
Unsatisfied Autonomy Needs	-0.217 (-0.319, -0.115)	-0.102 (-0.187, -0.020)
Unsatisfied Capacity Needs	-0.269 (-0.353, -0.186)	-0.148 (-0.204, -0.093)
Unsatisfied Social Support Needs	-0.199 (-0.306, -0.091)	-0.080 (-0.136, -0.024)
Unsatisfied Respect Needs	-0.240 (-0.344, -0.137)	-0.092 (-0.151, -0.033)
Mastery	-0.123 (-0.188, -0.059)	-0.030 (-0.075, 0.015)
Satisfied Basic Needs	-0.068 (-0.866, 0.730)	-0.031 (-0.765, 0.588)
Satisfied Security Needs	0.181 (0.043, 0.304)	0.015 (-0.377, 0.067)
Satisfied Autonomy Needs	0.027 (-0.178, 0.229)	0.024 (-0.136, 0.179)
Satisfied Capacity Needs	-0.030 (-0.206, 0.143)	-0.041 (-0.152, 0.067)
Satisfied Social Support Needs	-0.070 (-0.165, 0.026)	-0.054 (-0.106, -0.002)
Satisfied Respect Needs	-0.009 (-0.123, 0.101)	0.008 (-0.037, 0.051)
**Random Effects Coefficients**		
Bo	0.012 (0.000, 0.102)	0.000 (0.000, 0.006)
Bombali	0.001 (0.000, 0.008)	0.001 (0.000, 0.013)
Bonthe	0.003 (0.000, 0.041)	0.001 (0.000, 0.012)
Kailahun	0.001 (0.000, 0.009)	0.001 (0.000, 0.008)
Kambia	0.001 (0.000, 0.010)	0.000 (0.000, 0.004)
Koinadugu	0.003 (0.000, 0.037)	0.001 (0.000, 0.015)
Kono	0.000 (0.000, 0.002)	0.000 (0.000, 0.001)
Moyamba	0.001 (0.000, 0.015)	0.000 (0.000, 0.006)
Port Loko	0.000 (0.000, 0.003)	0.000 (0.000, 0.001)
Pujehun	0.001 (0.000, 0.015)	0.001 (0.000, 0.006)
Tonkolili	0.005 (0.000, 0.068)	0.000 (0.000, 0.004)
Western Rural	0.011 (0.000, 0.140)	0.004 (0.000, 0.050)
Western Urban	0.034 (0.000, 0.220)	0.002 (0.000, 0.027)
**Random Effects Parameters***		
Mean (μ)	0.208 (0.017, 0.663)	0.220 (0.019, 0.722)
Variance (σ^2)	1.368 (0.074, 4.217)	1.710 (0.133, 5.170)

* Used to parameterize $\Gamma(\alpha, \beta)$ where $\alpha = {}^{\mu^2}/_{\sigma^2}$ and $\beta = {}^{\mu}/_{\sigma^2}$.

T6. Coefficients for Hierarchical Bayesian Logistic Regression Model of Program Status

Covariable	Estimate (95% CrI)
Unsatisfied Basic Needs	-0.325 (-0.433, -0.217)
Unsatisfied Security Needs	-0.591 (-0.687, -0.496)
Unsatisfied Autonomy Needs	-0.167 (-0.294, -0.039)
Unsatisfied Capacity Needs	-0.653 (-0.768, -0.538)
Unsatisfied Social Support Needs	-0.740 (-0.876, -0.604)
Unsatisfied Respect Needs	-1.247 (-1.426, -1.071)
Mastery	0.388 (0.276, 0.502)
Satisfied Basic Needs	2.539 (0.498, 5.738)
Satisfied Security Needs	-0.062 (-0.246, 0.121)
Satisfied Autonomy Needs	1.033 (0.712, 1.357)
Satisfied Capacity Needs	1.240 (0.994, 1.486)
Satisfied Social Support Needs	-0.026 (-0.249, 0.198)
Satisfied Respect Needs	-0.088 (-0.277, 0.097)
Epi Week	0.000 (-0.002, 0.002)
**Random Effects Coefficients**	
Bo	0.117 (0.000, 0.261)
Bombali	0.527 (0.393, 0.662)
Bonthe	0.209 (0.000, 0.445)
Kailahun	0.004 (0.000, 0.029)
Kambia	0.008 (0.000, 0.056)
Koinadugu	0.007 (0.000, 0.048)
Kono	0.567 (0.403, 0.734)
Moyamba	0.011 (0.000, 0.071)
Port Loko	0.191 (0.057, 0.315)
Pujehun	0.003 (0.000, 0.018)
Tonkolili	0.851 (0.701, 1.003)
Western Rural	0.802 (0.530, 1.090)
Western Urban	0.078 (0.000, 0.310)
**Random Effects Parameters***	
Mean	0.495 (0.145, 1.315)
Variance	1.096 (0.284, 3.270)

* Used to parameterize distribution for random effects $\Gamma(\alpha, \beta)$ where $\alpha = {}^{\mu^2}/_{\sigma^2}$ and $\beta = {}^{\mu}/_{\sigma^2}$.
